# The methyltransferase MLL4 promotes nonalcoholic steatohepatitis by enhancing NF-κB signaling

**DOI:** 10.1016/j.jbc.2024.107984

**Published:** 2024-11-13

**Authors:** Junekyoung Lee, Hyejin An, Chong-Su Kim, Seunghee Lee

**Affiliations:** 1 Research Institute of Pharmaceutical Sciences, Natural Products Research Institute, College of Pharmacy, Seoul National University, Seoul, South Korea; 2 Department of Food and Nutrition, College of Natural Information Sciences, Dongduk Women's University, Seoul, South Korea

**Keywords:** epigenetics, histone methylation, NF-kappa B (NF-κB), inflammation, gene expression, hepatocyte, macrophage

## Abstract

Non-alcoholic fatty liver disease (NAFLD) is a growing health problem worldwide, ranging from non-alcoholic fatty liver (NAFL) to the more severe metabolic non-alcoholic steatohepatitis (NASH). Although many studies have elucidated the pathogenesis of NAFLD, the epigenetic regulatory mechanism from NAFL to NASH remains incompletely understood. The histone H3 lysine 4 methyltransferase, MLL4 (also called KMT2D), is a critical epigenetic transcriptional coactivator that mediates overnutrition-induced steatosis in mice, but its potential role in the progression of NASH remains largely unknown. Here, we show that mice lacking the one allele of the *Mll4* gene are resistant to hepatic steatosis, inflammation, and fibrosis in NASH conditions compared to wild-type controls. Transcriptome analysis of the livers of control and *Mll4*^*+/−*^ mice identified pro-inflammatory genes regulated by the nuclear factor kappa B (NF-κB) signaling pathway as major target genes of MLL4. We show that MLL4 binds to p65 and that MLL4 is required for NF-κB transactivation. Myeloid-specific *Mll4* knockout mice showed an almost complete block of NASH, while hepatocyte-specific *Mll4* knockout mice showed mild inhibition of steatosis. Pro-inflammatory M1 polarization is decreased and anti-inflammatory M2 polarization is increased in liver macrophages from myeloid-specific *Mll4* knockout mice. Importantly, we show that histone H3-lysine 4 methylation mediated by the MLL4-complex plays a critical role in promoting the expression of *Ccl2* in hepatocytes and M1 marker genes in macrophages. Our results demonstrate that MLL4, through the NF-κB-MLL4 regulatory axis, exacerbates steatohepatitis in the context of an inflammatory response and represents a potential therapeutic target for NASH.

Non-alcoholic fatty liver disease (NAFLD) has a high prevalence worldwide and encompasses a wide range of liver diseases, from simple steatosis to the more serious and progressive disease, non-alcoholic steatohepatitis (NASH) (1). NASH is characterized by overactivation of the hepatic immune system and tissue damage leading to fibrosis and ultimately cirrhosis, making it a leading cause of liver-related morbidity and mortality (2). However, therapeutic options are limited as our understanding of the mechanisms of NASH is incomplete (3).

NASH is likely the result of multiple hits, including metabolic steatosis and other cellular damage leading to inflammation, fibrosis, and hepatocellular death (4, 5). However, the molecular mechanisms and integration underlying the pathogenic process at different stages of liver disease are poorly understood. Extensive studies have shown that macrophages of the innate immune system, including liver-resident macrophages (Kupffer cells) and recruited monocyte-derived macrophages (MDMs), play an important role in the progression of NASH (6, 7, 8). In mouse dietary models such as high-fat diet (HFD) and methionine and choline-deficient (MCD) diet (MCDD), infiltration of F4/80^+^ macrophages and activation of Kupffer cells with increased expression of pro-inflammatory cytokines have been observed (5, 9).

Inflammatory chemokine (C-C motif) ligand 2 (Ccl2; also known as monocyte chemoattractant protein-1 or MCP-1) is known to attract monocytes and T cells during liver injury (10). Ccl2 is produced by many types of liver cells, including hepatocytes, stellate cells, and Kupffer cells (11, 12). The Ccl2-CCR2 signaling axis plays a central role in inflammation and the immune response. CCR2 is the primary receptor for Ccl2 and this axis is critical for the recruitment of monocytes and macrophages to sites of inflammation and tissue injury (13, 14). During acute or chronic liver injury, CCR2^+^ monocytes from the circulation are recruited to the liver in response to the Ccl2 and constitute the majority of hepatic macrophages that contribute to the pathogenesis of NASH (15, 16). Increased activation of Toll-like receptors (TLR) on non-parenchymal cells (NPCs), such as Kupffer cells and hepatic stellate cells (HSCs), leads to increased secretion of Ccl2 (17, 18) and promotes recruitment of inflammatory cells to the damaged liver (19) and regulates the wound healing response (20). In addition, Ccl2 secretion from hepatocytes represents an early response to parenchymal injury. Hepatocyte-specific *Ccl2* knockout mice showed reduced MDM infiltration and liver fibrosis. In contrast, forced hepatocyte Ccl2 expression in chow-fed wild-type mice induced the opposite phenotype, which was ameliorated by concomitant hepatocyte *Ccl2* knockout or CCR2 inhibitor treatment (21).

Macrophages can differentiate into opposing functional phenotypes, such as the classically activated pro-inflammatory M1 type or the alternatively activated anti-inflammatory M2 type (22). Classical M1 macrophages, activated primarily by endotoxin/lipopolysaccharide (LPS), induce a high inflammatory response and bactericidal effects, whereas alternative M2 macrophages, activated by Th2 cytokines (such as IL-4 and IL-13), promote resolution of inflammation and tissue remodeling (23). M1 liver macrophages exacerbate liver damage by producing inflammatory cytokines such as tumor necrosis factor-α (TNFα), but M2 liver macrophages can inhibit M1 macrophage activation by secreting anti-inflammatory cytokines such as IL-10 (22).

The expression of the pro-inflammatory cytokines such as TNFα and IL-1β is induced by activation of the toll-like receptor 4 (TLR4) and nuclear factor kappa B (NF-κB) pathways (24, 25, 26). The transcription factor NF-κB regulates multiple aspects of innate and adaptive immune function and serves as a central mediator of inflammatory responses (27, 28). In addition, macrophage polarization towards the M1 phenotype is mediated by NF-κB signaling, which promotes hepatic inflammation, and thus inhibition of the NF-κB pathway contributes to the protective effects of NASH (29, 30, 31).

Changes in epigenetic modifications contribute significantly to the development of NAFLD by altering transcriptional gene regulatory networks involved in lipid homeostasis, mitochondrial function, and inflammation (32, 33, 34). However, in particular, the histone modifications involved in the transition from simple steatosis to steatohepatitis remain unclear. The methylation pattern of the histone H3 lysine 4 residue creates flexible epigenetic marks that are strongly associated with gene induction (35, 36). While H3K4 mono- and di-methylations (H3K4me1 and H3K4me2) mark enhancers, H3K4 tri-methylation (H3K4me3) marks promoters (37, 38). In mammals, six Set1-like complexes contain one of the H3K4MTs, Set1A/B, mixed-lineage leukemia 1 to 4 (MLL1-4), complex-specific subunits, and a common subcomplex consisting of Rbbp5, Ash2l, Wdr5, and Dpy30 that facilitates H3K4MT activity. We have previously purified MLL3 (KMT2C) and MLL4 (KMT2D) complexes, which also contain the histone H3 lysine 27 (H3K27) demethylase UTX (35). Interestingly, MLL3/4 complexes have been shown to play important roles in several metabolic processes, in particular MLL4 as a critical regulator of overnutrition-induced hepatic steatosis (39, 40). However, the role of the MLL4 complex beyond the steatosis step has not yet been investigated and the identity of its target genes/enhancers associated with NASH progression as well as the transcription factors that recruit the MLL4 complex to these genes remain to be further defined.

Here, using a combination of the MCDD-induced NASH paradigm and unbiased genome-wide RNA sequencing (RNA-seq) analyses, our findings demonstrate that MLL4 acts as a critical epigenetic regulator in the progression of NASH by modulating the NF-κB signaling pathway in hepatocytes and macrophages, highlighting the important role of NF-κB in the inflammatory response associated with NASH. Overall, our results reveal a key function of MLL4 during NASH progression and represent a potential target for therapeutic development in steatohepatitis.

## Results

### Positive expression of MLL4 in NASH patients and protective effects of *Mll4* deletion on liver function

**Figure 1:**
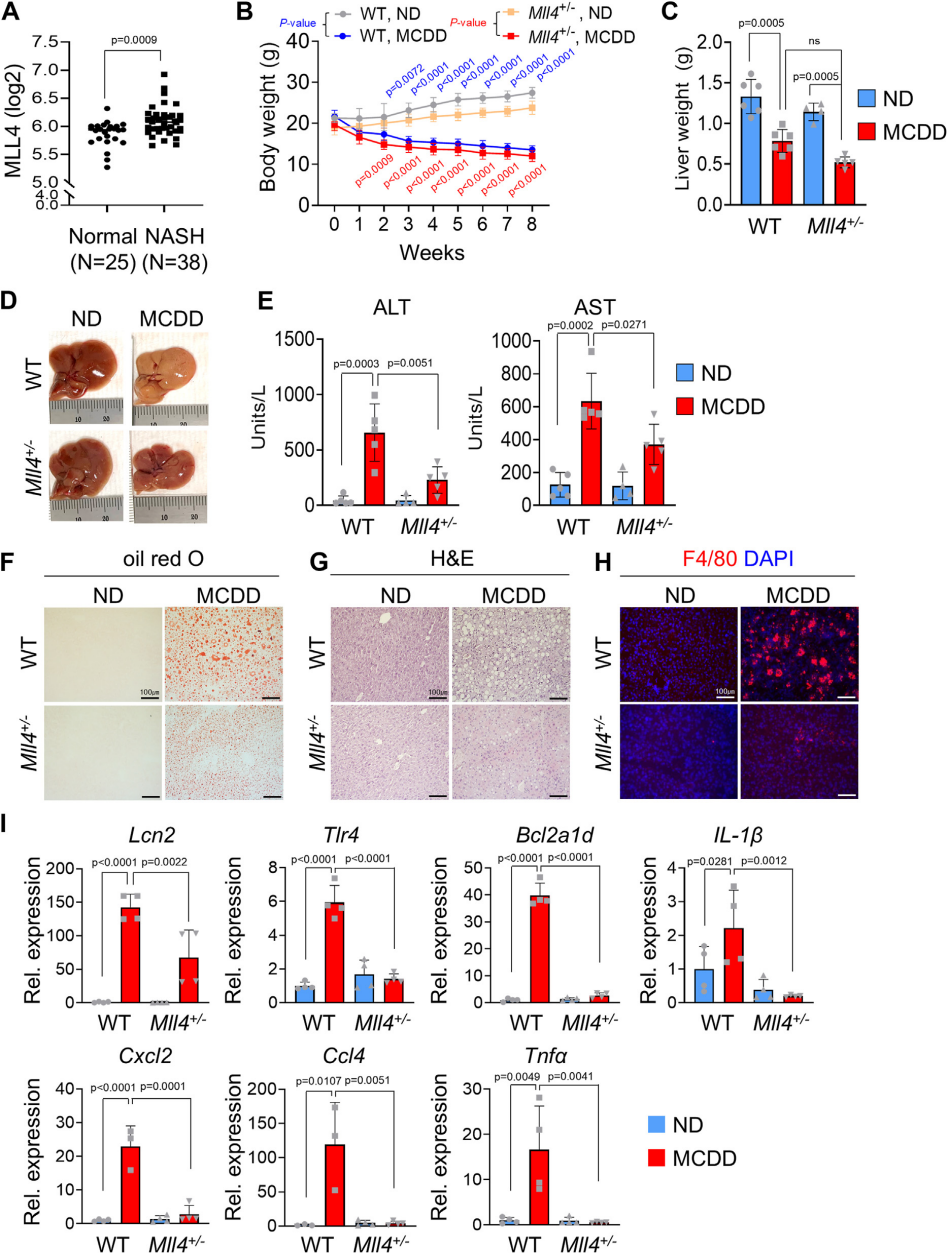
**MLL4 expression in NASH patients and NASH protection in *Mll4***^***+/−***^**mouse livers.***A*, *MLL4* gene expression analysis in liver tissues from NASH patients was conducted using GEO data sets (GSE48452 and GSE61260) from the NCBI database. Gene expression levels are presented on a log2 scale, comparing normal (n = 25) and NASH (n = 38) samples. *B*–*I*, eight-week-old WT and *Mll4*^+/−^ male mice were fed a normal diet (ND) or a methionine and choline-deficient diet (MCDD) for 8 weeks (n = 4–6 for each group). *B*, weakly body weights (g) were measured. MCDD-fed WT and *Mll4*^+/−^ mice showed a significant decrease in body weight compared to ND-fed mice. *C*, liver weights (g) were measured after 8 weeks of ND and MCDD feeding. *D*, gross morphology of livers from WT and *Mll4*^+/−^ mice was observed after 8 weeks of ND or MCDD feeding. *E*, serum transaminase levels (AST and ALT) were measured in WT and *Mll4*^+/−^ mice (n = 4–5 for each group). *F*, oil red O staining was performed on liver tissues to assess lipid accumulation. *G*, *H* & *E* staining of liver tissues was used to examine histological changes. *H*, immunofluorescence staining for F4/80 and DAPI was conducted on liver tissues from WT and *Mll4*^+/−^ mice-fed ND or MCDD. Scale bars represents 100 μm. *I*, qRT-PCR was used to analyze the expression of inflammation-associated genes in liver tissues by (n = 3–4 for each group). Results are presented as mean ± SD. Statistical differences were determined by two-sided Student’s *t* test (*A*) and two-way ANOVA with Tukey’s multiple comparisons test (*B*, *C*, *E*, and *I*). The exact *p*-values are reported in each graph.

To investigate the correlation with MLL4 expression in NASH patients, we analyzed gene expression in NASH patients using GEO datasets (GSE48452 and GSE61260) obtained from the NCBI database (41, 42). MLL4 expression was significantly increased in liver tissue from patients with NASH compared to normal livers (Fig. 1*A*), suggesting a role in the pathogenesis of NASH. As *Mll4*^*−/−*^ embryos died before embryonic day 9.5, we used *Mll4*^*+/−*^ mice for the study (43). To investigate whether lowering MLL4 levels leads to resistance to MCDD-induced steatohepatitis in mice, we subjected *Mll4*^*+/−*^ mice to MCDD for 8 weeks. We observed an approximate 40% reduction in body weight in both wild-type (WT) and *Mll4*^*+/−*^ mice following the MCDD (Fig. 1*B*) as previously reported (44). In terms of liver weight, both WT and *Mll4*^*+/−*^ mice showed a trend of reduction when subjected to the MCDD (Fig. 1*C*). While control mice displayed liver tissue with MCDD-induced yellowish steatohepatitis, *Mll4*^*+/−*^ mice were resistant to MCDD-induced steatohepatitis showing almost normal overall liver tissue (Fig. 1*D*). Alanine aminotransferase (ALT) and aspartate aminotransferase (AST) are enzymes found primarily in the liver. They play a crucial role in amino acid metabolism. Elevated levels of these enzymes in the blood can indicate liver damage or disease (45, 46, 47). *Mll4*^*+/−*^ mice showed decreased ALT and AST levels under MCDD feeding compared to WT mice (Fig. 1*E*). Histological analysis of *Mll4*^*+/−*^ mice treated with MCDD showed reduced steatosis in oil red O staining (Fig. 1*F*), consistent with our previous report on HFD condition (39). In addition, hematoxylin and eosin (H&E) staining revealed significantly less lobular infiltration of inflammatory cells and hepatocyte ballooning in the livers of *Mll4*^*+/−*^ mice compared to WT mice (Fig. 1*G*). Furthermore, immunofluorescence staining of liver sections with the macrophage-specific antibody F4/80 showed that deletion of a single copy of *Mll4* in mice significantly ameliorated MCDD-induced liver inflammation (Fig. 1*H*). Consistently, the expression of genes associated with MCDD-induced inflammation, such as *Lcn2, Tlr4, Bcl2a1d, Il1b, Cxcl2, Ccl4,* and *Tnfα*, was dramatically reduced in *Mll4*^*+/−*^ mice as measured by quantitative RT-PCR (qRT-PCR) (Fig. 1*I*). These findings suggest that MLL4 plays an important role in regulating pro-inflammatory gene expression and liver inflammatory pathology in NASH.

**Figure 2:**
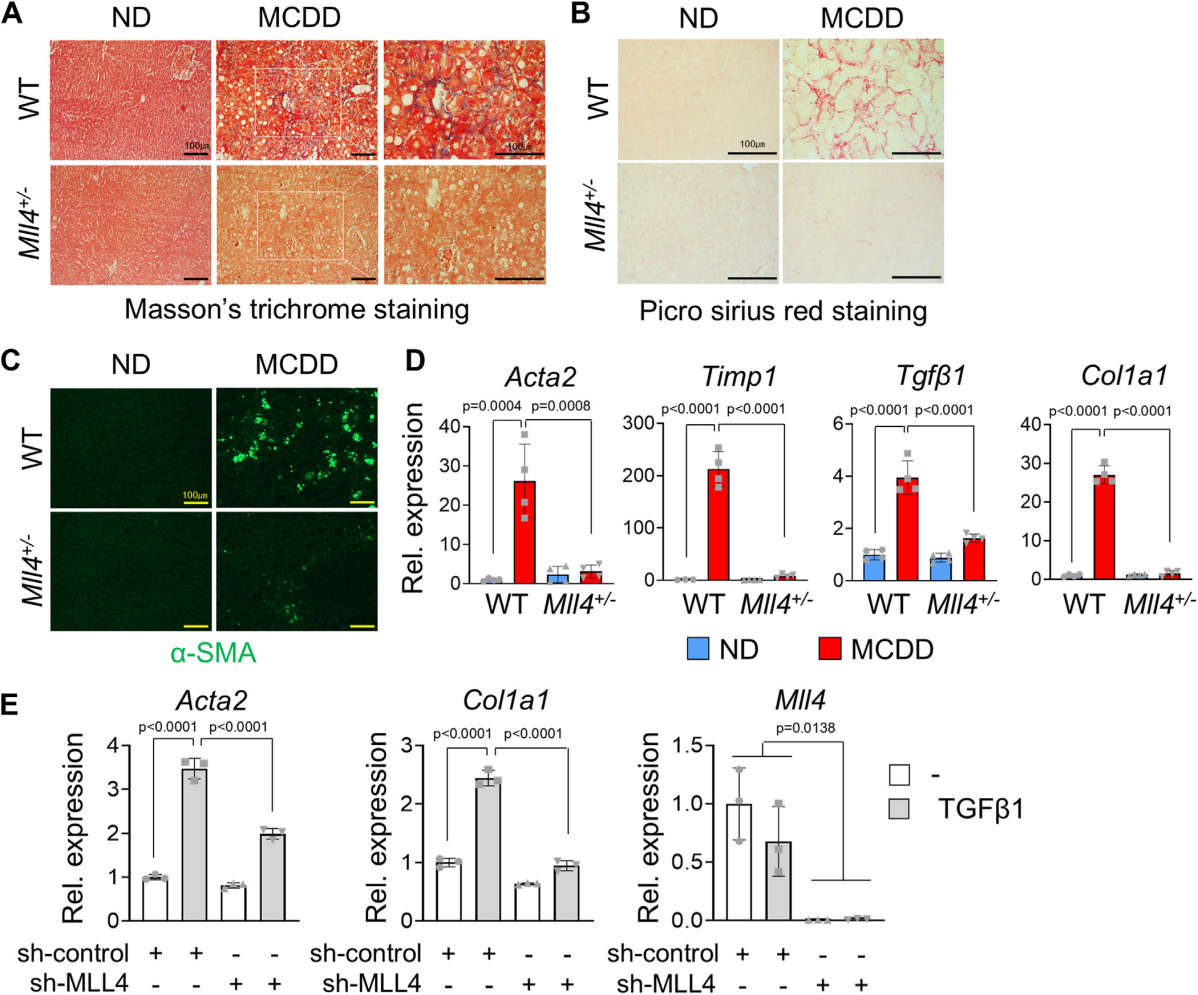
**Resistance of *Mll4***^***+/−***^**mice to MCDD-induced liver fibrosis.***A*, Masson’s trichrome staining was performed on liver tissues to assess fibrosis. *B*, Picro Sirius Red staining was used to evaluate collagen deposition in liver tissues. *C*, immunofluorescence (IF) analysis for α–SMA was conducted on liver sections from WT and *Mll4*^+/−^ mice-fed ND or MCDD. Scale bars represents 100 μm. *D*, expression levels of fibrosis-associated genes in liver tissues were measured by qRT-PCR (n = 3–4 for each group). *E*, treatment of LX2 cells with TGFβ1 (8 ng/ml) for 24 h elevated fibrosis-associated gene expression, whereas MLL4 knockdown reduced their expression, as measured by qRT-PCR. Data represent the mean ± SD from experiments performed in triplicate. Statistical differences were determined using two-way ANOVA with Tukey’s multiple comparisons test. The exact *p*-values are reported in each graph.

Next, we investigated whether MCDD-induced liver fibrosis could be ameliorated by deletion of a single copy of *Mll4* in mice. Alanine blue is typically used in Masson’s trichrome staining to highlight collagen fibers and Sirius red staining also detects collagen, particularly type I and type III collagen fibers. Staining of liver sections with Masson's trichrome (Trichr) and Sirius red (Sir red) for liver collagen showed decreased staining for aniline blue and Sirius red in *Mll4*^*+/−*^ mice compared to MCDD-fed WT mice (Fig. 2, *A* and *B*). Staining of alpha-smooth muscle actin (α-SMA) for activated myofibroblast-like HSCs also decreased in *Mll4*^*+/−*^ mice compared to MCDD-fed WT mice (Fig. 2*C*). These results suggest less liver fibrosis development upon deletion of a single copy of *Mll4*. At the mRNA level, *Mll4* deletion caused a robust reduction in the expression of genes associated with fibrosis, including *Acta2 (α-SMA), Timp1, Tgfβ1, and Col1a1* as measured by qRT-PCR (Fig. 2*D*). To further investigate the role of MLL4 in liver fibrosis, we performed MLL4 knockdown in human LX2 hepatic stellate cells, which are widely used to model fibrogenesis. LX2 cells were transfected with a small hairpin RNA (shRNA) construct against MLL4 (sh-MLL4) to reduce MLL4 expression and then treated with TGF*β*1, a potent inducer of fibrosis. Following treatment, we assessed the expression of fibrosis-related genes, including *Acta2* and *Col1a1*, using qRT-PCR. Our results demonstrated that the knockdown of MLL4 significantly impaired the induction of these genes (Fig. 2*E*). These results suggest a critical role for MLL4 in promoting fibrosis in NASH.

### Critical role of MLL4 in MCDD-dependent transcriptome changes in mouse liver and identification of inflammatory target genes of MLL4

**Figure 3:**
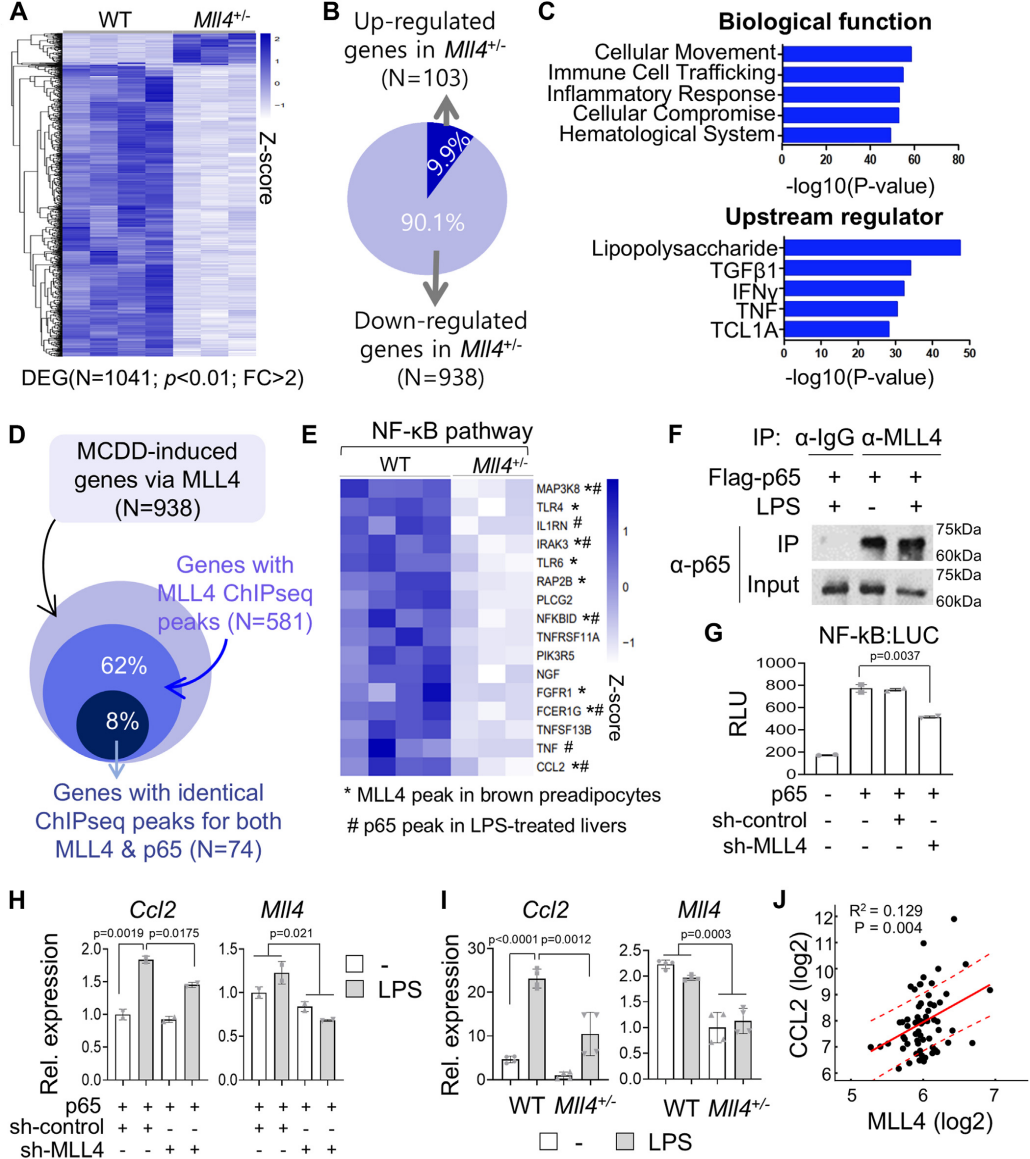
**MLL4 target genes in NASH formation.***A*, heatmap showing differentially expressed MLL4 target genes under NASH-inducing conditions (MCDD-fed WT: n = 4, MCDD-fed *Mll4*^+/−^: n = 3). *B*, schematic illustrating that a significant portion of MCDD-regulated genes are MLL4-dependent. *C*, top significant biological functions and upstream regulators of differentially expressed genes in *Mll4*^+/−^ livers. The *p*-value was calculated using a right-tailed Fisher’s Exact test. *D*, schematic representation showing that a portion of MCDD-induced genes contain ChIP-seq peaks for both MLL4 and p65. *E*, heatmap clustering of MCDD-induced MLL4 target genes involved in the NF-κB pathway for NASH formation. An asterisk (∗) denotes MLL4 peaks in brown preadipocytes, and a hash (#) denotes p65 peaks in LPS-treated livers. *F*, co-immunoprecipitation (CoIP) of MLL4 and p65 in HepG2 cells. HepG2 cells expressing Flag-p65 were subjected to immunoprecipitation with an anti-MLL4 antibody, followed by immunoblotting with an anti-p65 antibody. This revealed that MLL4 interacts with p65, and this interaction is enhanced by LPS (100 ng/ml) treatment for 2 h. *G*, Hepa1c1c7 cells were transfected with NF-κB:luciferase reporter and expression vectors for p65, sh-control, or sh-MLL4. Luciferase activity was normalized to β-galactosidase activity. Transfections were repeated independently at least three times. Data are shown as relative luciferase units (RLU) (mean ± SD). *H*, qRT-PCR results indicated that *Ccl2*, an NF-κB target gene, was induced by LPS (100 ng/ml) treatment for 6 h, but this induction was suppressed by MLL4 knockdown in Hepa1c1c7 cells. The reduced expression of MLL4 by sh-MLL4 was confirmed by qRT-PCR. *I*, *Ccl2* expression in primary hepatocytes from WT mice was induced by LPS (100 ng/ml) treatment for 6 h but not in hepatocytes from *Mll4*^*+/−*^ mice (n = 2 for each group). *J*, public datasets from the NCBI GEO database (http://www.ncbi.nlm.nih.gov/geo/) were analyzed for gene expression correlation between MLL4 and CCL2 using NASH patient GEO datasets (GSE48452 and GSE61260). The correlation coefficient (r^2^) was calculated using Pearson’s correlation test. The x- and y-values in Figure 3*J* are log2-transformed. The red solid line represents the regression line, and the red dashed line indicates one standard error bounds. Results are presented as mean ± SD. Statistical differences were determined by two-sided Student’s *t* test (*G*) and two-way ANOVA with Tukey’s multiple comparisons test (*H* and *I*). Exact *p*-values are reported in each graph.

Based on the strong protective effects of *Mll4* mutants against MCDD-induced liver dysfunction and the fact that MLL4 acts as a transcriptional coactivator, we hypothesized that MLL4 may play an important role in MCDD-dependent transcriptome changes in the mouse liver. To test this hypothesis, we performed RNA-seq analysis on the livers of eight-week-old WT and *Mll4*^*+/−*^ mice fed the MCDD for eight weeks. These analyses revealed that a total of 1041 genes were significantly altered between the two groups, with 938 genes (more than 90%) downregulated and 103 genes upregulated in the livers of *Mll4*^*+/−*^ mice (fold change>2.0, *p* < 0.01) (Fig. 3, *A* and *B*). Gene ontology (GO) analysis of 938 suppressed genes in the liver of *Mll4*^*+/−*^ mice revealed significantly altered biological functions, including inflammatory response, immune cell trafficking, and cellular movement (Fig. 3*C*). Interestingly, the top five upstream regulators that were differentially regulated in the liver of *Mll4*^*+/−*^ mice were identified by functional analysis of RNA-seq data, including lipopolysaccharide (LPS), TGFβ1, IFNγ, and TNF, which are known NASH-promoting factors (Fig. 3*C*).

### NF-κB as a key mediator of the pro-inflammatory function of MLL4

From the RNA-seq data analysis, we found that one of the pathways significantly downregulated in the livers of *Mll4*^*+/−*^ mice compared to wild-type mice was the NF-κB signaling pathway, with LPS, which activates this pathway, identified as a key upstream regulator. In addition, ASC-2 (NCOA6), a transcriptional coactivator and a critical component of the MLL4 complex, has previously been reported to interact with p65 (RELA) and enhance the transcriptional activity of p65 (48). This interaction is likely to contribute to the recruitment of the MLL4 complex to NF-κB target genes. To test this idea, we examined the reported chromatin immunoprecipitation sequencing (ChIP-seq) peaks for MLL4 and p65 (49, 50). Among 938 MCDD-induced genes by MLL4, 581 genes (62%) showed ChIP-seq peaks for MLL4, and 74 genes (8%) showed ChIP-seq peaks for both MLL4 and p65 (Fig. 3*D*). Nine of the sixteen genes involved in the NF-κB pathway had ChIP-seq peaks for MLL4 (indicated by ∗ in Fig. 3*E*), and 5 of these genes had ChIP-seq peaks for both MLL4 and p65 (indicated by ∗ and # in Fig. 3*E*).

We then tested whether MLL4 could interact with p65 using a coimmunoprecipitation (CoIP) assay in HepG2 cells transfected with flag-tagged p65. Immunoprecipitation with anti-MLL4 antibody pulled down p65. This interaction was enhanced in the presence of LPS, suggesting that LPS treatment triggers transcriptional activation of NF-κB by recruiting the MLL4 complex to its target genes (Figs. 3*F* and S5). To test whether the transcriptional activity of NF-κB is affected by MLL4 knockdown in cells, we performed the luciferase reporter assay using NF-κB:LUC, which contains the NF-κB binding site. The activity of the luciferase reporter activated by p65 expression was inhibited by co-transfected small hairpin RNA (shRNA) construct against MLL4 (sh-MLL4), but not by the control shRNA vector (Fig. 3*G*). These results suggest that NF-κB is an important transcription factor that recruits the MLL4 complex to the MCDD-induced target genes that promote NASH formation.

### Transactivation of hepatic target genes of the MLL4 complex by NF-κB in liver and selective regulation of Ccl2 by MLL4 in hepatocytes

To independently validate the RNA-seq results, we performed qRT-PCR for the genes involved in the NF-κB pathway on liver tissue from MCDD-fed WT and *Mll4*^*+/−*^ mice. The expression levels of *Map3k8, Il1rn, Irak3, Nfkbid, Tnfα*, and *Ccl2* were significantly suppressed in the livers of *Mll4*^*+/−*^ mice compared to control WT mice (Fig. S1). Interestingly, in the mouse hepatocarcinoma cell line Hepa1c1c7, LPS-induced *Ccl2* expression was significantly impaired by MLL4 knockdown (sh-MLL4), while the expression of other NF-κB target genes remained unaffected (Figs. 3*H* and S2*A*). Similarly, in primary hepatocytes isolated from *Mll4*^*+/−*^ mice, LPS treatment resulted in a marked reduction of *Ccl2* expression without influencing other genes (Figs. 3*I* and S2*B*). These findings demonstrate that MLL4 specifically regulates *Ccl2* expression in hepatocytes as a transcriptional co-activator of NF-κB signaling. Furthermore, analysis of NASH patient datasets (GSE48452 and GSE61260) revealed a positive correlation between MLL4 and CCL2 expression (Fig. 3*J*), highlighting the selective regulatory role of MLL4 on *Ccl2*.

### Mild suppression of NASH in hepatocyte-specific MLL4-deleted mice

**Figure 4:**
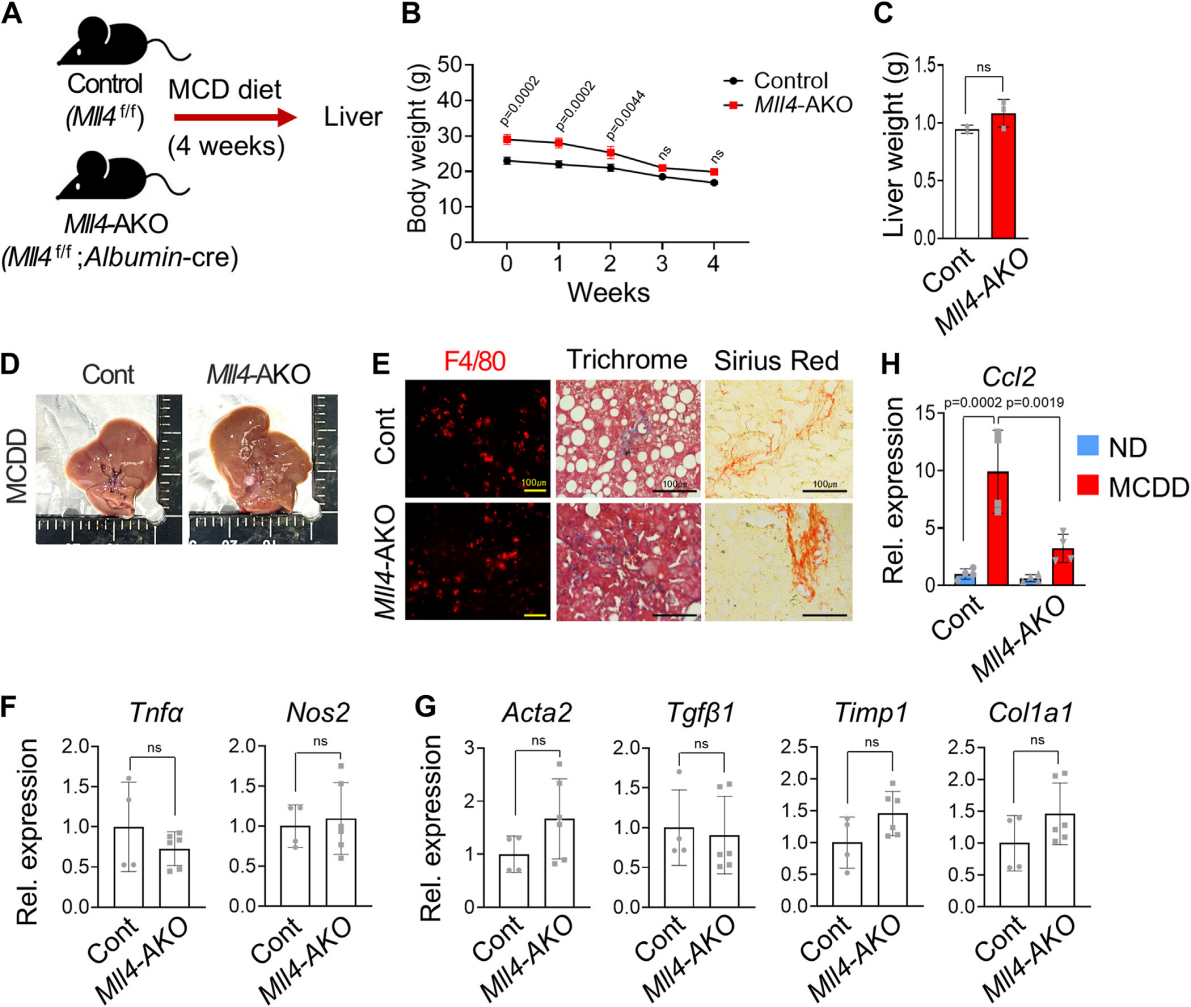
**Mild inhibition of NASH in hepatocyte-specific MLL4-deleted mice.***A*, eight-week-old *Mll4*^f/f^ (Control) (n = 2) and *Mll4*^f/f^; *Albumin*-cre (*Mll4*-AKO) (n = 3) mice were fed an MCDD for 4 weeks. *B* and *C*, body weight (g) and liver weight (g) of *Mll4*^f/f^ (n = 2) and *Mll4*-AKO (n = 3) mice after 4 weeks of MCDD feeding. *D*, gross liver morphology from WT and *Mll4*-AKO mice sacrificed after 4 weeks of MCDD. *E*, IF analysis for F4/80, Masson’s trichrome staining, and Picro Sirius Red staining of liver tissues from *Mll4*^f/f^ and *Mll4*-AKO mice-fed MCDD. Scale bars represents 100 μm. *F* and *G*, expression of pro-inflammatory genes and fibrosis-associated genes in liver tissues from *Mll4*^f/f^ and *Mll4*-AKO mice-fed MCDD (n = 2–3 for each group). *H*, expression of *Ccl2* in liver tissues from *Mll4*^f/f^ and *Mll4*-AKO mice-fed ND or MCDD, as measured by qRT-PCR (n = 2 for each group). Results are presented as mean ± SD. Statistical differences were determined by two-sided Student’s *t* test (*C*, *F* and *G*) and two-way ANOVA with Tukey’s multiple comparisons test (*B* and *H*). The exact *p*-values are reported in each graph.

To further dissect the cell types in which MLL4 functions to promote NASH, we first generated hepatocyte-specific *Mll4* knockout mice, the *Mll4*^f/f^; *Albumin*-Cre (*Mll4*-AKO), by crossing *Mll4*^f/f^ mice with *Mll4*^f/+^;*Albumin*-Cre mice. *Mll4*-AKO and control *Mll4*^f/f^ littermates were then subjected to MCDD for 4 weeks (Fig. 4*A*), expecting that NASH resistance in *Mll4*-AKO would be similar to that in *Mll4*^*+/−*^ mice. There were no differences in body weight and liver weight between MCDD-fed *Mll4*^f/f^ and *Mll4*-AKO mice (Fig. 4, *B* and *C*). Surprisingly, *Mll4*-AKO mice still develop steatohepatitis compared to control littermates (Fig. 4*D*). Histological analysis of F4/80, trichrome, and Sirius red staining showed that MCDD-induced liver inflammation and fibrosis were not significantly blocked by *Mll4* deletion in hepatocytes (Fig. 4*E*). In addition, the expression levels of pro-inflammatory genes, such as *Tnfα* and *Nos2*, and fibrosis-related genes, such as *Acta2, Tgfβ1, Timp1, and Col1a1,* were not reduced in the livers of *Mll4*-AKO mice compared to controls under MCDD conditions (Fig. 4, *F* and *G*).

However, *Mll4*-AKO livers exhibited reduced lipid accumulation and inhibited steatogenic gene expression, aligning with observation in *Mll4*^*+/−*^ mice (Fig. S3, *A* and *B*). Consistent with the findings in Figure 3, *H* and *I*, *Ccl2* expression was significantly impaired in the livers of *Mll4*-AKO mice (Fig. 4*H*), indicating that hepatocyte-specific *Mll4* deletion impairs *Ccl2* expression. Nonetheless, this reduction alone was insufficient to prevent NASH progression, suggesting that MLL4 in other cell types is involved in NASH pathogenesis. Therefore, while MLL4 in hepatocytes plays a role in regulating adipogenic gene expression under conditions of overnutrition or NASH and Ccl2-mediated macrophage recruitment, its deletion in hepatocytes alone does not fully protect against NASH.

### Reduction of liver inflammation and fibrosis by deletion of *Mll4* in Kupffer cells/macrophages

**Figure 5:**
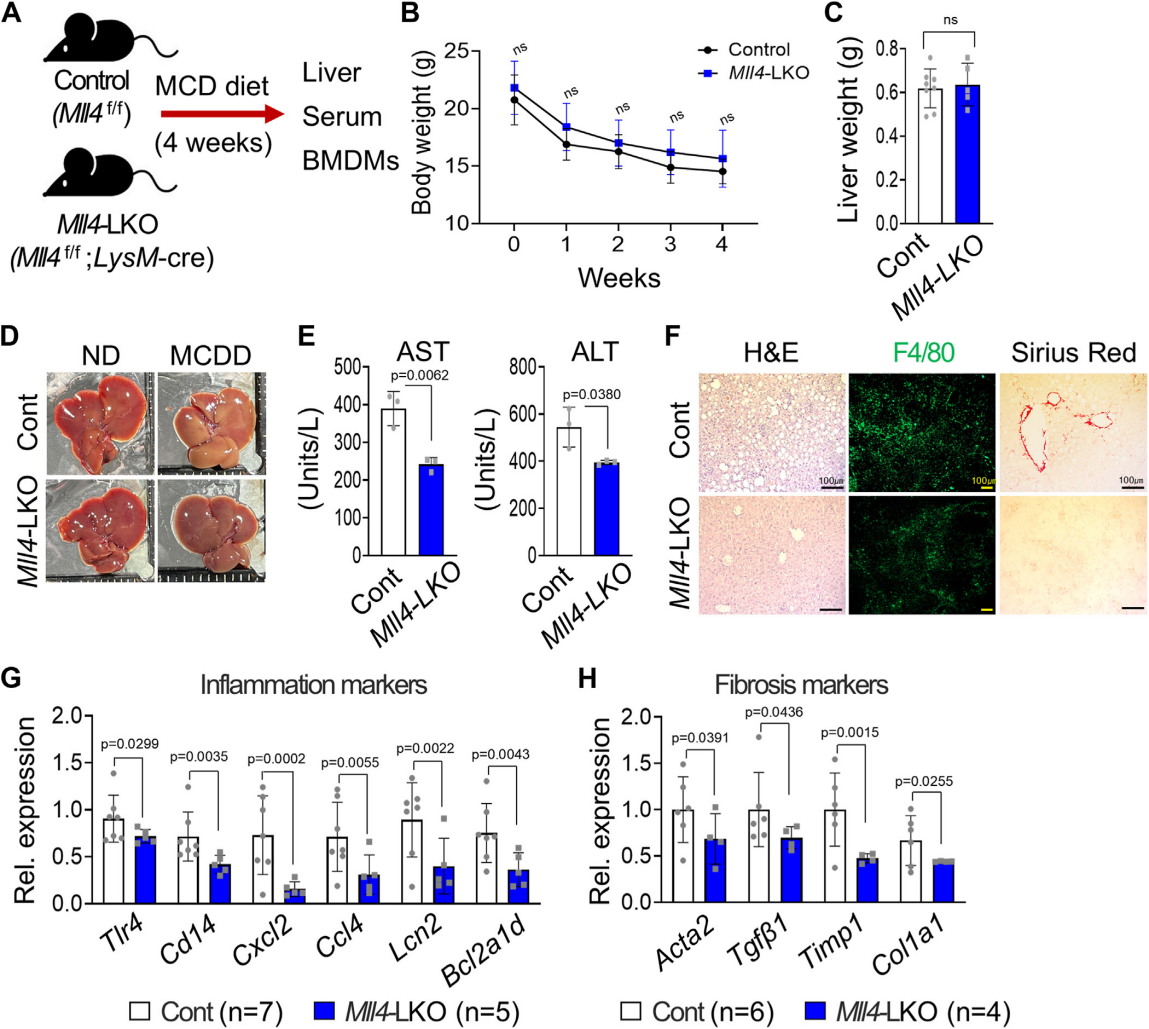
**Deletion of MLL4 in KCs/macrophages reduces NASH.***A*, eight-week-old *Mll4*^f/f^ (Control) (n = 8) and *Mll4*^f/f^;*LysM*-cre (*Mll4*-LKO) (n = 5) mice were fed with MCDD for 4 weeks. Schematic outline of experimental approaches and analysis. *B* and *C*, body weight (g) and liver weight (g) of *Mll4*^f/f^ (n = 8) and *Mll4*-LKO (n = 5) mice after 4 weeks of MCDD feeding. *D*, representative captured liver tissues of experimental mice at the end of experiments. *E*, levels of serum transaminases (AST and ALT) measured from *Mll4*-LKO (n = 3) and *Mll4*^f/f^ mice (n = 3) fed MCDD. *F*, *H* & *E* staining, IF analysis for F4/80, and Sirius Red staining of liver tissues from *Mll4*^f/f^ (n = 6) and *Mll4*-LKO (n = 3) fed MCDD. Scale bars represents 100 μm. *G* and *H*, qRT-PCR analysis for inflammation- and fibrosis-associated genes in liver tissues from MCDD-fed *Mll4*^f/f^ (n = 6–7) and *Mll4*-LKO (n = 4–5). Results are presented as mean ± SD. Statistical differences were determined by two-sided Student’s *t* test (*C*, *E*, *G* and *H*) and two-way ANOVA with Tukey’s multiple comparisons test (*B*). The exact *p*-values are reported in each graph.

To test whether MLL4 expressed in macrophages plays an important role in NASH development, we next generated myeloid cell-specific *Mll4* knockout mice, the *Mll4*^f/f^; *LysM*-Cre (*Mll4*-LKO), by crossing *Mll4*^f/f^ mice with mice carrying a *Mll4*^f/+^;*LysM*-Cre transgene. *Mll4*-LKO and control *Mll4*^f/f^ littermates were then subjected to MCDD for 4 weeks (Fig. 5*A*). There were no differences in body weight and liver weight between MCDD-fed *Mll4*^f/f^ and *Mll4*-LKO mice (Fig. 5, *B* and *C*). It was evident that the overall morphology and color of the liver in *Mll4*-LKO were normal even in the MCDD condition (Fig. 5*D*). The levels of AST and ALT were also lower in *Mll4*-LKO mice than in control littermates under MCDD (Fig. 5*E*).

Histological analysis revealed less lobular inflammation in the liver tissue of *Mll4*-LKO compared to control mice. Furthermore, F4/80 and Sirius red staining of liver sections showed that the specific deletion of *Mll4* in macrophages significantly ameliorated MCDD-induced liver inflammation and fibrosis (Fig. 5*F*). Similar to *Mll4*^*+/−*^ mice, the induction of inflammation and fibrosis marker genes by MCDD was significantly suppressed in *Mll4*-LKO mice (Fig. 5, *G* and *H*). These findings suggest that MLL4, expressed in Kupffer cells (KCs)/liver macrophages, plays a critical role in promoting inflammation and fibrosis during NASH formation by upregulating pro-inflammatory and fibrotic genes.

### Decrease in pro-inflammatory M1 phenotype and increase in anti-inflammatory M2 phenotype in *Mll4*-LKO mice

**Figure 6:**
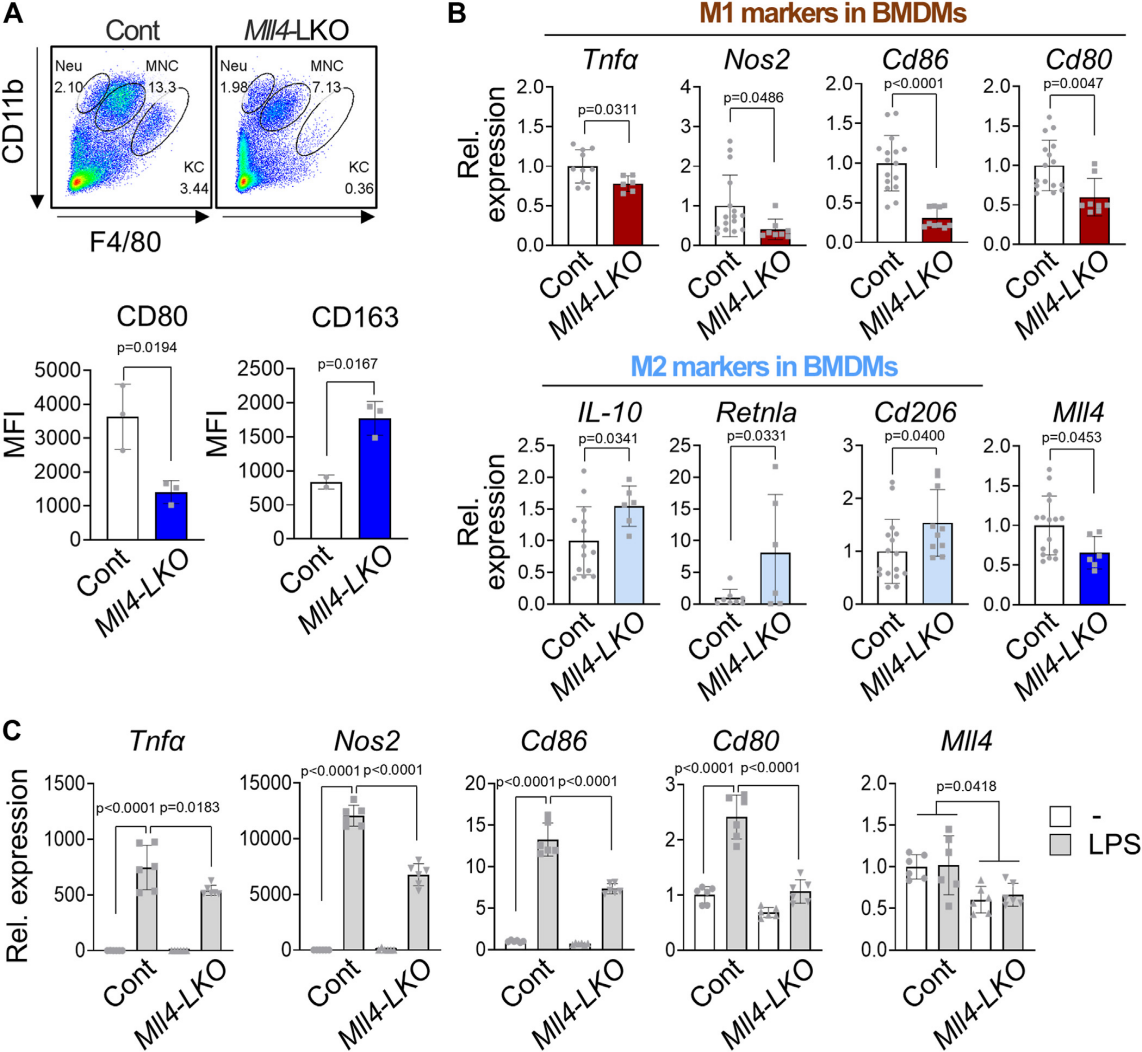
**Macrophage/monocyte-specific MLL4 deletion inhibits M1 phenotype.***A*, flow cytometry analysis of primary Kupffer cells (KCs) from *Mll4*^f/f^ (n = 3) and *Mll4*-LKO (n = 3) mice fed an MCDD for 4 weeks. Bottom: Mean fluorescence intensity (MFI) of CD80^+^ M1 KCs and CD163^+^ M2 KCs was measured. *B*, expression of M1 and M2 markers in primary bone marrow–derived macrophages (BMDMs) from MCDD-fed *Mll4*^f/f^ (n = 4–8) and *Mll4*-LKO (n = 3–5) mice, determined by qRT-PCR. *C*, M1 marker expression in primary BMDMs from ND-fed *Mll4*^f/f^ (n = 3) and *Mll4*-LKO (n = 3) mice treated with LPS (100 ng/ml) for 6 h, assessed by qRT-PCR. Results are shown as mean ± SD. Statistical differences were determined by two-sided Student’s *t* test (*A* and *B*) and two-way ANOVA with Tukey’s multiple comparisons test (*C*). The exact *p*-values are reported in each graph.

To test the involvement of MLL4 in M1/M2 polarization in the liver, we next examined the differences in the immune responses of KCs between *Mll4*-LKO and control mice in the MCDD condition. Flow cytometry analysis showed that F4/80-positive KCs were decreased in MCDD-fed *Mll4*-LKO mice (Fig. 6*A*). This analysis also showed that CD80-positive M1 KCs were decreased and CD163-positive M2 KCs were increased in MCDD-fed *Mll4*-LKO mice compared to control mice (Fig. 6*A*). We isolated bone marrow-derived myelocytes (BMDMs) from the livers of MCDD-fed *Mll4*-LKO and control mice and examined the expression profile of M1 and M2 marker genes. The expression of M1 markers such as *Tnfα*, *Nos2, Cd86,* and *Cd80* was significantly decreased, whereas the expression of M2 markers such as *IL-10, Retnla,* and *Cd206* was increased in BMDMs from *Mll4*-LKO mice compared to BMDMs from control mice (Fig. 6*B*). Since M1 polarization of KCs is known to be mediated by the NF-κB pathway, we investigated whether the LPS-induced M1 polarization is inhibited in *Mll4*-deficient BMDMs. We isolated primary BMDMs from *Mll4*-LKO and control mice and treated them with LPS for 6 hours. M1 markers such as *Tnfα*, *Nos2, Cd86, and Cd80* were highly induced by LPS treatment in control BMDMs but not in *Mll4*-LKO BMDMs (Fig. 6*C*). These results suggest that MLL4 functions as a key transcriptional activator that induces the expression of pro-inflammatory M1 genes through LPS-dependent NF-κB transactivation, which promotes NASH progression.

### Transactivation of NASH-promoting genes by the MLL4 complex *via* NF-κB in the liver

**Figure 7:**
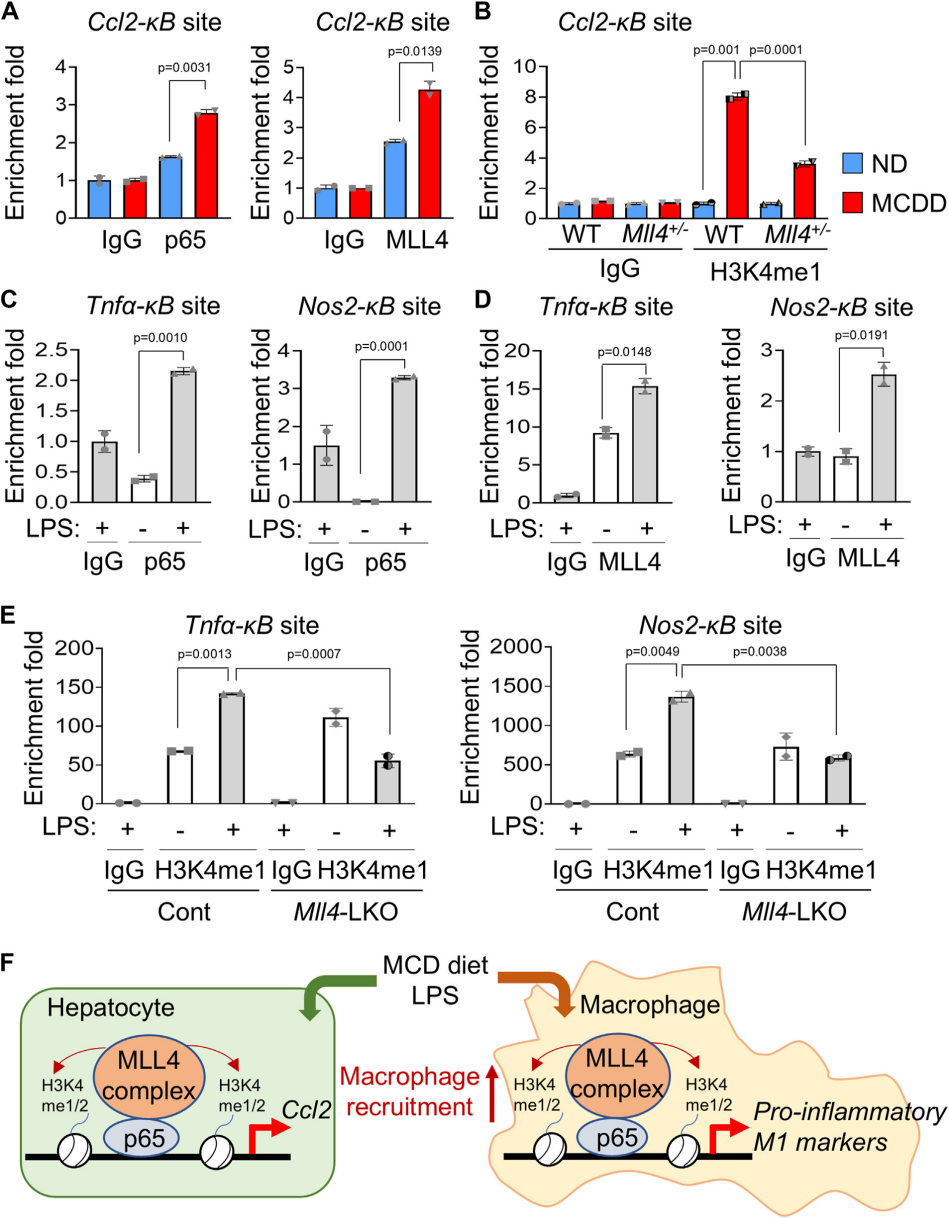
**Critical roles of MLL4 in NF-κB target gene expression in the liver.***A*, chromatin immunoprecipitation (ChIP) analysis showing MCDD-enhanced recruitment of p65 and MLL4 to the *Ccl2-κB* site in primary hepatocytes from WT mice-fed ND or MCDD. *B*, ChIP results displaying elevated H3K4me1 levels at the *Ccl2-κB* site in primary hepatocytes from WT livers under MCDD, but not in primary hepatocytes from *Mll4*^+/−^ livers. *C* and *D*, ChIP for LPS-enhanced recruitment of p65 and MLL4 to the *Tnfα-κB* and *Nos2-κB* sites in primary BMDMs from WT mice. *E*, ChIP showing increased H3K4me1 levels at the *Tnfα-κB* and *Nos2-κB* sites in primary BMDMs from *Mll4*^f/f^, but not from *Mll4*-LKO. Results are shown as mean ± SD. Statistical differences were evaluated using a two-sided Student’s *t* test (*A*, *C*, and *D*) and two-way ANOVA with Tukey’s multiple comparisons test (*B* and *E*). The exact *p*-values are reported in each graph. *F*, working model: MLL4 plays a key role in NASH development. MCDD or LPS triggers activation of the NF-κB-MLL4 axis, promoting the decoration of NF-κB target genes with H3K4me1/2 marks in the liver. This leads to the transactivation of *Ccl2* in hepatocytes, which recruits macrophages to damaged liver sites. In macrophages, MLL4 drives the transcription of inflammatory, fibrotic, and M1 marker genes, exacerbating NASH progression.

To investigate whether NF-κB and MLL4 are indeed responsible for the transcriptional activation of the *Ccl2* gene, we found the NF-κB response element (*κB* site) from the ChIP-seq peak for both MLL4 and p65, located approximately 3kb upstream of the transcription start site (indicated by the red box in Fig. S4). We tested whether p65 and MLL4 are recruited to the *Ccl2-κB* site by ChIP assay using control IgG, anti-p65, and anti-MLL4 antibodies in primary hepatocytes from normal diet (ND)- and MCDD-fed WT mouse livers. Indeed, we found that recruitment of p65 and MLL4 to the *Ccl2-κB* site was enhanced in the MCDD-fed condition (Fig. 7*A*), which coincided with an increase in H3K4me1 levels in the primary hepatocytes from MCDD-fed WT mice (Fig. 7*B*). Furthermore, H3K4me1 levels were significantly dampened in primary hepatocytes from MCDD-fed *Mll4*^*+/−*^ mice (Fig. 7*B*), demonstrating that the decoration of this *Ccl2-κB* region by H3K4me1 is performed by MLL4. These results suggest that NF-κB plays a critical role in recruiting the MLL4 complex to the *Ccl2* gene and that MLL4 in turn directs H3K4me1 modification of the *Ccl2* enhancer region in hepatocytes.

We also identified MLL4 binding peaks using ChIP-seq data from bone marrow cells (51, 52), which overlap with p65 binding regions in the intron regions of the *Tnfα* and *Nos2* genes (indicated by red boxes in Fig. S4). These regions contain *κB* sites. Furthermore, using ChIP assays on primary BMDMs isolated from WT mice, we verified the LPS-dependent recruitment of both p65 and MLL4 to these regions. As expected, p65 was recruited to the *κB* sites of the *Tnfα* and *Nos2* genes upon treatment with LPS (100 ng/ml) for 6 h (Fig. 7*C*), and MLL4 recruitment to these *kB* sites was also increased in the presence of LPS (Fig. 7*D*). Furthermore, H3K4me1 levels induced by LPS treatment in control BMDMs were significantly attenuated in *Mll4*-LKO BMDMs at both *Tnfα*-*κB* and *Nos2-κB* sites (Fig. 7*E*). These results suggest that the NF-κB-MLL4 regulatory axis plays a critical role in promoting M1 polarization with the induction of pro-inflammatory target genes in response to LPS or MCDD in liver macrophages.

## Discussion

MLL4 has been reported to be associated with hepatic steatosis in previous studies (39). However, it has remained speculative whether MLL4 plays a role in the progression of NASH. In this study, we provide evidence that macrophage-expressed MLL4 is a critical player in promoting steatohepatitis in MCDD-induced NASH mouse models. These data suggest a potentially novel non-parenchymal function of MLL4, coupled with its role in lipogenesis and Ccl2 induction in hepatocytes (Fig. 7*F*).

Under NASH-driving conditions, *Mll4* heterozygous mutant mice were resistant to liver steatosis, inflammation, and fibrosis, suggesting that MLL4 is a critical factor in the progression of liver disease (Figs. 1 and 2). Using unbiased RNA-seq analysis, we found that the inflammatory response genes regulated by LPS, TGFβ1, IFNγ, and TNF require MLL4 for expression (Fig. 3*C*), establishing MLL4 as an important player in MCDD-induced transcriptome changes in the mouse liver. In addition, our RNA-seq results suggest that the NF-κB pathway is a key mediator in recruiting the MLL4 complex to the MCDD- or LPS-regulated target genes of MLL4, including genes involved in liver inflammation and fibrosis (Fig. 3, *D* and *E*).

Although Ccl2 is known to be a major contributor to the recruitment of liver monocyte-derived macrophages (MDMs) to sites of liver injury and to promote fibrosis, the relative contribution of hepatocyte-derived Ccl2 to NASH pathogenesis and the underlying mechanism of *Ccl2* expression in hepatocytes is relatively unknown. Recently, it has been reported that Notch-induced Ccl2 expression in hepatocytes is both necessary and sufficient for liver infiltration of MDMs and NASH-induced liver fibrosis (21). Interestingly, we found that *Ccl2* is one of the MLL4 target genes mediated by NF-κB activation in the hepatocytes. *Ccl2* expression levels were significantly suppressed in the livers of *Mll4*^*+/−*^ mice compared to control WT mice under MCDD conditions (Fig. S1). *Ccl2* expression induced by LPS was impaired in the MLL4 knockdown mouse liver cell line Hepa1c1c7 as well as in primary mouse liver hepatocytes (Fig. 3, *H* and *I*). Furthermore, *Ccl2* expression was reduced in the livers of hepatocyte-specific *Mll4-*deleted mice (Fig. 4*H*). These results suggest that MLL4 in hepatocytes functions to induce chemokine expression through H3K4 methylation in the NF-κB binding region such as the *Ccl2* gene locus (Fig. 7*B*). It is also possible that other transcription factors, such as the Notch intracellular domain/mastermind-like/recombination signal binding protein for immunoglobulin kappa J region (NICD/MAML/Rbpj) transcription complex, recruit the MLL4 complex to the *Ccl2* promoter. Future studies should test this possibility and identify additional transcription factors that mediate liver inflammation and fibrosis *via* the MLL4 complex in the liver.

The mild inhibition of MCDD-induced inflammation in hepatocyte-specific *Mll4* deficient mice suggests that MLL4 expressed in other cell types plays a critical role in the exacerbation of NASH. Indeed, myeloid cell-specific *Mll4* knockout mice were resistant to the development of MCDD-induced NASH (Fig. 5). In addition, M1 KCs were reduced in MCDD-fed *Mll4*-LKO mice compared to control mice and, in parallel, the expression of M1 markers was significantly reduced in BMDM from *Mll4*-LKO mice compared to control mice (Fig. 6). As for the underlying molecular mechanism of how M1 marker gene expression is regulated by MLL4, we also showed that transactivation of these genes is mediated by NF-κB activation by LPS (Fig. 7, *C* and *D*). Increased H3K4me1 levels induced by LPS treatment in control BMDMs were significantly attenuated in *Mll4*-LKO BMDMs at NF-κB and MLL4 target gene loci (Fig. 7*E*). These results suggest that the NF-κB-MLL4 regulatory axis in liver macrophages plays an important role in promoting M1 polarization with the induction of inflammatory target genes (Fig. 7*F*).

The epigenetic regulation of NASH is poorly understood. Our identification of the NF-κB-MLL4 axis provides critical insights into this issue. Through this axis, NASH-inducing conditions such as MCDD or LPS treatment lead to inflammation and fibrosis by mobilizing the MLL4 complex to the hepatic inflammatory and M1 polarizing target genes of NF-κB-MLL4, which in turn facilitates their open chromatin formation. In particular, we have shown that recruitment of the MLL4 complex leads to an increase in the open chromatin mark H3K4me1 (Fig. 7, *B* and *E*). It remains to be determined whether UTX in the MLL4 complex also plays an important role in NASH progression *via* its ability to remove the repressive chromatin mark H3K27me3, which is essential to establish the active enhancer mark H3K27ac.

Previously, we have shown that the treatment with imatinib, originally developed as a specific inhibitor of BCR-ABL, ameliorates the fatty liver condition of *ob/ob* and HFD-fed mice, at least in part, by interfering with the interactions of PPARγ 2 with the MLL4 complex and antagonizing the recruitment of MLL4 to steatotic target genes of MLL4 (39). A recent study showed that Imatinib reduced pro-inflammatory markers in murine macrophages *in vitro* and in obese mouse models *in vivo* by targeting SREBP1, which links lipid metabolism and the innate immune response, but did not affect the PPARγ phosphorylation at serine 273 (53). The dampened SREBP transcriptional programs by imatinib preceded the restoration of PPARγ-phosphorylation-related genes in adipose tissue, suggesting that restored PPARγ-phosphorylation may be a secondary phenomenon. In particular, imatinib inhibited TNFα production *in vitro* and prevented TNF-dependent acute liver inflammation (54). In addition, imatinib treatment in patients with chronic myeloid leukemia (CML) has shown a significant downregulation of NF-κB activation (55). Overall, imatinib has a preferential immunosuppressive effect in macrophages, while in the liver it also affects lipogenesis, suggesting the multiple effects of imatinib mediated by PPARγ, NF-κB, and SREBP pathways. It should be noted that imatinib also inhibits the phosphorylation of substrate proteins, including the inhibitory protein IκB, which keeps the NF-κB complex in an inactive state in the cytoplasm (54).

In summary, this study identifies a critical epigenetic regulatory axis in NASH progression and we also present this NF-κB-MLL4 axis as a potential target for anti-NASH drug development. Any small molecule that antagonizes the H3K4MT enzymatic activity of MLL4 may ameliorate both NASH and fatty liver disease in NAFLD patients. Our findings extend previous literature by linking MLL4 activity not only to lipogenesis, but also to innate immunity in the context of NAFLD. A deeper understanding of the integrated pathways between inflammation and lipid metabolism may pave the way for the development of novel therapeutics in NAFLD.

## Experimental procedures

### Animals

All mouse work was performed with the approval of the Institutional Animal Care and Use Committee of Seoul National University. Mice were housed in the animal facility under a standard 12h light/12h dark cycle. Mice had *ad libitum* access to water and were fed normal diet (ND) and methionine and choline-deficient diet (MCDD) (22% fat, 8.2 g/kg methionine, 1.4 g/kg choline, TD.90262, ENVIGO) for up to 8 weeks. The diet lacks methionine and choline, which are essential for very-low-density lipoprotein (VLDL) synthesis and lipid export from the liver, leading to hepatic steatosis, inflammation, and fibrosis (44, 56). Previous studies have described *Mll4*^+/−^ and *Mll4*^f/f^ mice (43, 49). To generate hepatocyte and myeloid cell-specific *Mll4*-deleted mouse lines (*Mll4*-AKO *and Mll4*-LKO), *Mll4*^f/f^ mice were crossed with *Albumin*-Cre or *LysM*-Cre mice (The Jackson Laboratories), producing *Mll4*^f/+^;*Albumin*-Cre or *Mll4*^f/+^;*LysM*-Cre mice respectively. These mice were next crossed with *Mll4*^f/f^ mice to generate *Mll4*^f/f^;*Albumin*-Cre (*Mll4*-AKO) or *Mll4*^f/f^;*LysM*-Cre (*Mll4*-LKO) mice. Mouse genotypes were determined by PCR using mouse tail genomic DNA. Primer sequences used for genotyping are listed below. Blood was collected from the heart of the mouse, and only the supernatant (blood plasma) was centrifuged at 4 °C and analyzed using an automatic blood analyzer, Fuji DRI-Chem 3500s (FUJIFILM Corporation, Tokyo, Japan). All animal experiments were performed after approval by the Institutional Animal Care and Use Committee of the Institute of Laboratory Animal Resources, Seoul National University (Institutional Animal Care and Use Committee approval number: SNU-240527–3). All experiments were performed in accordance with the ARRIVE guidelines and regulations.

### Plasmids

NF-κB:LUC has been described previously (57, 58). Flag-tagged mouse p65 gene was cloned into a pcDNA3 vector for mammalian expression. For knockdown of MLL4 in mouse Hepa1c1c7 cells and human LX2 cells, short hairpin RNA (shRNA) constructs against mouse and human MLL4 were prepared in EFU6-300 vector, which contain GFP to monitor transfected cells. The shRNA-targeting sequences were as follow: 5′-GCA AGG TGC CTC GAG ATA A-3′ and 5′-TCG CAT GCG TTG CCC CAA T-3′ for mouse MLL4 and human MLL4, respectively.

### Cell culture and luciferase reporter assay

All cells were cultured in a humidified chamber with 5% CO_2_ at 37 °C and tested monthly for *mycoplasma* contamination using the e-Myco plus PCR Detection Kit (iNtRON). HepG2 cells (HB-8065, ATCC), a human liver cancer cell line, were cultured in DMEM High Glucose (L0103, Biowest) supplemented with 10% heat-inactivated fetal bovine serum (FBS) and 1% penicillin/streptomycin. Hepa1c1c7 cells (CRL-2026, ATCC), a murine hepatoma cell line, were cultured in MEM alpha (12561056, Gibco) supplemented with 10% FBS and 1% penicillin/streptomycin. LX2 cells (SCC064, Sigma), a human hepatic stellate cell line, were cultured in DMEM High Glucose supplemented with 2% heat-inactivated FBS and 1% penicillin/streptomycin. Hepa1c1c7 cells and LX2 cells were seeded in 6-well plates and incubated for 24 h, followed by transient transfection using Lipofectamine 2000 (11668019, Thermo Fisher Scientific). Cells were harvested 48 h post-transfection after treatment with LPS (L2880, Sigma) for 6 h or TGFβ1 (CA59, Bon Opus Biosciences) for 24 h. For the luciferase reporter assay, Hepa1c1c7 cells were seeded in 48-well plates and incubated for 24 h, and transient transfections were performed using Lipofectamine 2000 (11668019, Thermo Fisher Scientific). NF-κB:LUC reporter, Flag-p65, sh-control or sh-Mll4, and actin promoter β-galactosidase plasmid were transfected. Cell extracts were analyzed for luciferase activity and values normalized to β-galactosidase activity. Data are presented as the mean of duplicate values from represented experiments. Luciferase reporter assays were repeated independently at least three times. Data were shown in relative luciferase units (mean ± SD).

### Co-immunoprecipitation and immunoblotting

Flag-p65 plasmid was transfected into HepG2 cells by the superfect (QIAGEN) method and treated with 100 ng/ml LPS (L2880, Sigma) for 2 h before cell harvest. Cells were lysed in tissue lysis buffer (200 mM NaF, 20 mM Na-pyroPO4, 200 mM Tris-HCL, pH 8.0; 150 mM NaCl; 4 mM Na_3_VO_4_; 1 mM EDTA, pH8.0; 0.5% NP-40; 10% Glycerol; 2 mM PMSF), followed by immunoprecipitation with home-made α-MLL4 antibody and protein A-agarose beads (15918014, Thermo Fisher Scientific). The beads were then dissolved in SDS loading buffer by boiling at 100 °C for 5 min, and proteins were separated on sodium dodecyl sulfate polyacrylamide (SDS-PAGE) gels and transferred to nitrocellulose blotting membranes (Amersham Protran 02. μm NC, #10600001; GE Healthcare Life Science). The membranes were blocked for 1 h in 10% skim milk blocking solution (232100, BD Difco) and incubated with primary antibody against p65 (ab16502, Abcam) o/n at 4 °C. The next day, the membranes were incubated with secondary antibodies and developed with ECL solution (Clarity Max Western ECL substrate, #170562; BIO-RAD) on X-ray film. We have included Fig. S5, which provides a summary of all the original, uncropped immunoblots, with molecular weight markers clearly positioned at the appropriate locations.

### Isolation of primary hepatocytes, BMDMs, and Kupffer cells

For isolation of primary hepatocytes, liver tissue from 8-week-old mice was perfused with digestion buffer containing 0.5 mM EGTA (Invitrogen) and 1 mg/ml collagenase (C9891, Sigma-Aldrich), as described (59). The suspension cells were filtered through a 70 μm cell strainer and centrifuged at 50*g* for 5 min. Hepatocytes were then seeded onto a collagen-coated plate (Corning). Primary bone marrow-derived macrophages (BMDMs) were isolated from femoral and tibial bone marrow. Primary Kupffer cells were isolated from mouse livers by perfusion with collagenase (C9891, Sigma-Aldrich) as described (59). They were isolated by density gradient centrifugation using Optiprep (1114542, Axis-Shield/Serumwerk). The non-parenchymal cell fraction was resuspended in 20% Optipre. A layer of 11.5% Optiprep and HBSS (14175–095, Gibco) was applied to the suspended cells and centrifuged at 1811*g* for 17 min. Kupffer cells were extracted from a layer containing 11.5% Optiprep and 20% Optiprep. Primary BMDMs and Kupffer cells were plated and cultured in RPMI 1640 medium (SH30255.01, Cytiva) supplemented with 10% FBS and 1% penicillin/streptomycin. BMDMs were differentiated into macrophages with 20 ng/ml M-CSF (315–02, Peprotech) for 6 days. Cells were cultured in a serum-free medium for at least 12 h prior to LPS treatment and then 100 ng/ml LPS (L2880, Sigma) was added in a serum-free medium.

### Flow cytometry

To determine the M1/M2 status, Kupffer cells were stained with eF506-live/dead dye, FITC-F4/80, PE-CD80, APC-CD163 (eBioscience), and BV711-CD11b (BD bioscience) antibodies after incubation with FcII/III receptor antibodies (eBioscience). Kupffer cells were stained with Foxp3/Transcription Factor Staining Buffer Set (00–5523–00, eBioscience). Stained cells were analyzed using a high-end performance flow cytometer, LSRFortessa X-20 cell analyzer (BD bioscience) and Cell Quest software (BD bioscience).

### Immunofluorescence, oil red O, H&E, Masson’s trichrome, Picro Sirius Red staining

The right outer lobes of the livers were frozen in OCT solution, followed by sectioning on a cryostat at a thickness of 5 μm per section. Immunofluorescence staining was performed by incubating liver sections with α-F4/80 antibody (Cl:A3-1, Biorad) and α-SMA antibody (ab5694, Abcam) in blocking buffer o/n at 4 °C. The next day, the sections were reacted with the secondary fluorescent antibodies for 2 h at room temperature and then visualized and analyzed using a Zeiss fluorescence microscope. Frozen mouse liver slices cut at 5 μm were fixed in 4% paraformaldehyde and stained with 0.5% Oil Red O solution in propylene glycol for 30 min. For paraffin blocks, the left outer lobes of the livers were fixed in 10% formalin and embedded in paraffin. Paraffin-embedded livers were sectioned using a microtome at a thickness of 4 μm per section. The sectioned livers were then stained with hematoxylin and eosin (H&E), Masson's trichrome, and Picro Sirius Red according to standard protocols (60, 61).

### RNA-seq experiments and data analysis

All RNA from liver tissue was isolated using TRIzol (Thermo Fisher Scientific) and RNA samples were treated with DNase (Invitrogen) and then purified using RNeasy mini kit (Qiagen). RNA integrity was validated using a bioanalyzer. RNA-seq libraries were generated according to the TruSeq Stranded mRNA Sample Preparation Guide (Part # 15031047 Rev. E). RNA-seq libraries were validated by bioanalyzer and real-time RT-PCR. The NovaSeq6000 system was used to sequence the RNA-seq libraries. All base call files were converted to fastq format and used for analysis. Mapped sequencing reads were generated for the mouse reference genome (NCBI mm10) using the Top Hat and Bowtie algorithm. Raw read counts were normalized to Fragments Per Kilobase Million (FPKM) using Partek Genomics Suite v6.6 software (Partek, St Louis, MI) to quantify mRNA levels. Normalized counts were mapped according to each transcription. The differentially expressed genes (DEGs) with expression changes greater than 2.0-fold (*p* < 0.01) were included in the analysis. The DEGs were then used for downstream analyses including hierarchical clustering analysis and functional analysis. Biological functional analysis and upstream regulators identification of DEGs were performed using Ingenuity Pathway Analysis (IPA) and Database for Annotation, Visualization, and Integrated Discovery (DAVID) (62).

### RNA isolation and quantitative RT-PCR

All RNA from liver tissue was isolated using TRIzol (Thermo Fisher Scientific) and converted to cDNA using the Super Script III First-Strand Synthesis System (Invitrogen). Quantitative RT-PCR (qRT-PCR) was performed using SYBR Green qPCR Premix (RT500U, Enzynomics) on a Bio-Rad real-time PCR detection system. The relative gene expression values were normalized to *Cyclophilin A* by comparative Ct method. Relative expression levels were calculated from separate experimental replicates. All qRT-PCR values in this study are presented as the mean ± SD.

### ChIP and ChIP-seq analysis

ChIP assays with mouse livers and BMDMs were performed as described (63). The antibodies used for ChIP assays were α-IgG (sc-2027, Santa Cruz), α-H3K4me1 antibody (ab8895, Abcam), α-p65 antibody (ab16502, Abcam) and α-MLL4 antibody (HPA035977, Sigma). For ChIP-seq analysis, the ChIP-seq data used for analysis were obtained from the Gene Expression Omnibus (GEO) database under the CC0 Public Domain Dedication (http://www.ncbi.nlm.nih.gov/geo/). The corresponding accession numbers were GSE50466 and GSE103508 (MLL4 ChIP-seq), and GSE117488 and GSE211676 (p65 ChIP-seq). ChIP-seq data and peaks were displayed using the Integrated Genomics Viewer (IGV).

### Analysis of gene expression data from human NASH tissue samples

We retrieved datasets from the GEO database hosted by the National Center for Biotechnology Information (NCBI). Specifically, datasets GSE48452 and GSE61260 were selected for analysis as they provide comprehensive patient histories and key pathological parameters related to NASH, including steatosis, inflammation, and fibrosis. Notably, patients who had undergone bariatric surgery were excluded from the analysis (41, 42, 64).

### Quantification and Statistical analysis

Data values in this study are indicated as the mean ± SD. Statistical analysis was performed using Graph Pad Prism software (version 9.5.1). Unpaired Student’s *t*-tests were used to determine the differences between the two groups. The differences among multiple groups were analyzed by two-way ANOVA, followed by Tukey’s multiple comparisons test. Significance was defined by a *p*-value < 0.05.

ChIP primers used in this study

**Table:** 

Gene	Nucleotide sequence	Species
*TNFα-κB* site	(F) TGAGTTGATGTACCGCAGTCAAGA	Mouse
	(R) AGAGCAGCTTGAGAGTTGGGAAGT	
*Nos2-κB* site	(F) ATGGCCTTGCATGAGGATAC	Mouse
	(R) GGGCCAGAGTCTCAGTCTTC	
*Ccl2-κB* site	(F) TGGAAATTCCCATTCTGAGG	Mouse
	(R) GGCTGGGGATTGATGTTCTA	

qRT-PCR primers used in this study

**Table:** 

Gene	Nucleotide sequence	Species
*Lcn2*	(F) GCCAGTTCACTCTGGGAAAT	Mouse
	(R) GGTGGGGACAGAGAAGATGA	
*Tlr4*	(F) GCTTTCACCTCTGCCTTCAC	Mouse
	(R) GAAACTGCCATGTTTGAGCA	
*Bcl2a1d*	(F) GCCCTGGATGTAGGTGCTTA	Mouse
	(R) AAATGGAAATGCCAAGTGCT	
*IL-1β*	(F) GACCTTCCAGGATGAGGACA	Mouse
	(R) AGGCCACAGGTATTTTGTCG	
*Cxcl2*	(F) CAGACTCCAGCCACACTTCA	Mouse
	(R) TCAGGGTCAAGGCAAACTT	
*Ccl4*	(F) GCCCTCTCTCTCCTCTTGCT	Mouse
	(R) GCTGCTCAGTTCAACTCCAA	
*Tnfα*	(F) CAAATGGCCTCCCTCTCAT	Mouse
	(R) CATCGGCTGGCACCACTA	
*Acta2*	(F) CCTGACGGGCAGGTGATC	Mouse
	(R) ATGAAAGATGGCTGGAAGAGAGTCT	
*Acta2* *Timp1*	(F) TGGAAAAGATCTGGCACCAC (R) CGTCCAGAGGCATAGAGAGA (F) ATTCAAGGCTGTGGGAAATG	Human Mouse
	(R) CTCAGAGTACGCCAGGGAAC	
*Tgfβ1*	(F) TTGCTTCAGCTCCACAGAGA	Mouse
	(R) TGGTTGTAGAGGGCAAGGAC	
*Col1a1*	(F) GAGCGGAGAGTACTGGATCG	Mouse
*Col1a1*	(R) GCTTCTTTTCCTTGGGGTTC (F) ACATCAGCAAGAACCCCAAG (R) TGTTCTTGCAGTGGTAGGTG	Human
*α-SMA*	(F) CGTGGCTATTCCTTCGTGAC	Mouse
	(R) AGGTGGTTTCGTGGATGC	
*CD14*	(F) GAGTTGTGACTGGCCCAGTCAGC	Mouse
	(R) GCAAAGCCAGAGTTCCTGAC	
*CD80*	(F) CCATGTCCAAGGCTCATTCT	Mouse
	(R) TTCCCAGCAATGACAGACAG	
*CD86*	(F) CAGTGCTGGCAAATCAAGAA	Mouse
	(R) TTGCACAGCATTCTCCAGAC	
*Nos2*	(F) AATCTTGGAGCGAGTTGTGG	Mouse
	(R) CAGGAAGTAGGTGAGGGCTTG	
*IL-10*	(F) GCTCTTACTGACTGGCATGAG	Mouse
	(R) CGCAGCTCTAGGAGCATGTG	
*Retnla*	(F) CCCTGCTGGGATGACTGCTA	Mouse
	(R) TGCAAGTATCTCCACTCTGGATCT	
*CD206*	(F) CAGGTGTGGGCTCAGGTAGT	Mouse
	(R) TGTGGTGAGCTGAAAGGTGA	
*Pparg*	(F) AAGAAGCGGTGAACCACTGA	Mouse
	(R) CAGCAACCATTGGGTCAGCTC	
*Cpt1*	(F) CCAGGCTACAGTGGGACATT	Mouse
	(R) GAACTTGCCCATGTCCTTGT	
*Cd36*	(F) TCATGCCAGTCGGAGACATG	Mouse
	(R) TGTCTGTACACAGTGGTGCCTGT	
*Map3k8*	(F) GGCCCATGAGAGAATTTGAA	Mouse
	(R) GATGTCGGCTTTTGTGGAAT	
*Il1rn*	(F) GCTCATTGCTGGGTACTTACAA	Mouse
	(R) CCAGACTTGGCACAAGACAGG	
*Irak3*	(F) GTTCTAACGGGCTGCAAAGT	Mouse
	(R) TACAAGCTAGGCTGGGTGCT	
*Nfkbid*	(F) ATTTCCCCTGGTGATGGAGG	Mouse
	(R) GTCTCCTTCCTCATCCTGGG	
*Ccl2*	(F) AGGTCCCTGTCATGCTTCTG	Mouse
	(R) TCTGGACCCATTCCTTCTTG	
*CyclophilinA*	(F) GTCTCCTTCGAGCTGTTTGC	Mouse, Human
	(R) GATGCCAGGACCTGTATGCT	
*Mll4*	(F) CGAAGAACTCTTTGGGCTGACAGTG	Mouse
*Mll4*	(R) CGTTTATAGTGTGTGAGGATTTTCG (F) GCTCACAGTCTCTGCTGCTG (R) CAAACTGCTTCAGCCAATCA	Human

Mouse genotyping primers used in this study

**Table:** 

Gene	Nucleotide sequence	Species
P1+P3: *Mll4* WT	(P1) CGAAGAACTCTTTGGGCTGACAGTG	Mouse
P1+P2: *Mll4*^+/−^	(P2) CGCTCTTACCAAAGGGCAAACC	
	(P3) ATCTGCATCTCAAACCCTCAGAAGG	
*Mll4*^f/f^	(F) ATTGCATCAGGCAAATCAGC	Mouse
	(R) GCAGAAGCCTGCTATGTCCA	
*Albumin*-Cre	(F) GCGGTCTGGCAGTAAAAACTATC	Mouse
	(R) GTGAAACAGCATTGCTGTCACTT	
*LysM*-Cre	(F) CCCAGAAATGCCAGATTACG	Mouse
	(R) CTTGGGCTGCCAGAATTTCTC	

## Data availability

The RNA-sequencing dataset have been deposited in GEO (GSE277545). All data supporting the findings of this study are available within the article. Any further information is available from the corresponding author (leeseung@snu.ac.kr) upon request.

## Supporting information

This article contains supporting information (51, 52).

## Conflict of interests

The authors declare that they have no conflicts of interest with the contents of this article.
