# Supplementary material for: The methyltransferase MLL4 promotes nonalcoholic steatohepatitis by enhancing NF-κB signaling
Source: J Biol Chem. 2024 Nov 13;300(12):107984. doi: 10.1016/j.jbc.2024.107984 (PMC11665687; doi:10.1016/j.jbc.2024.107984)
Supplement: Supporting information [file mmc1.docx]

**Supporting information**

**The methyltransferase MLL4 promotes non-alcoholic steatohepatitis by enhancing NF-κB signaling**

Junekyoung Lee^1^, Hyejin An^1^, Chong-Su Kim^2^ and Seunghee Lee^1,^*

^1^ Research Institute of Pharmaceutical Sciences, Natural Products Research Institute, College of Pharmacy, Seoul National University, Seoul 08826, Korea,

^2^ Department of Food and Nutrition, College of Natural Information Sciences, Dongduk Women's University, Seoul 02748, Korea

**Running title:** MLL4 in NASH development

**Material Included:**

Figure S1. Validation of MLL4 target genes in the NF-κB pathway.

Figure S2. Expression of NF-κB pathway genes in Hepa1c1c7 cells and primary mouse hepatocytes.

Figure S3. Hepatocyte-specific deletion of *Mll4* prevents MCDD-induced steatosis.

Figure S4. A Schematic representation of ChIP-seq peaks for MLL4 and p65 at the *Ccl2, Tnfα* and *Nos2* loci.

Figure S5. Interaction between MLL4 and p65 in HepG2 cells.

**Supplementary Figures**


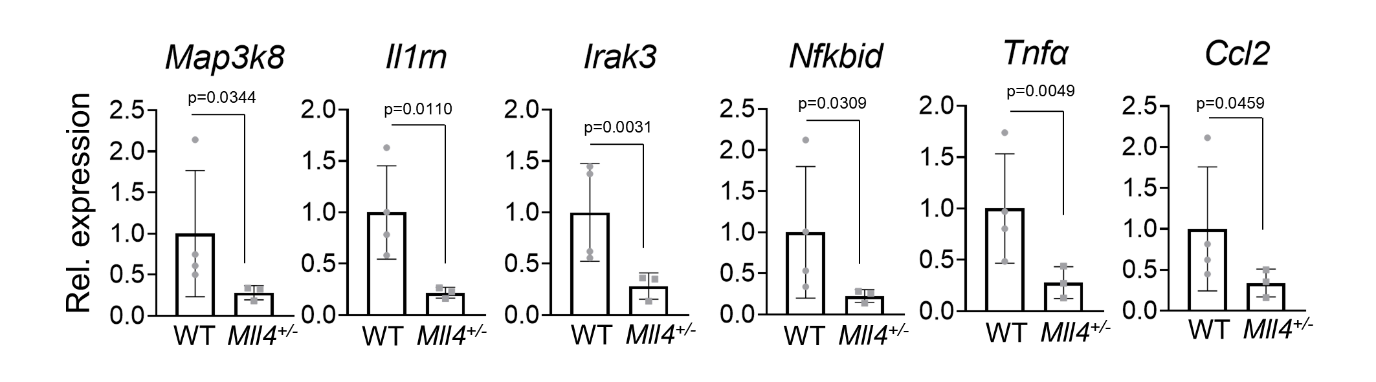


**Figure S1. Validation of MLL4 target genes in the NF-κB pathway.** qRT-PCR was performed to confirm MLL4 target genes identified by RNA-seq analysis in the livers of MCDD-fed WT (n=4) and *Mll4^+/-^* (n=3). Data represent the mean ± SD. Statistical differences were determined by two-sided Student’s *t*-test.


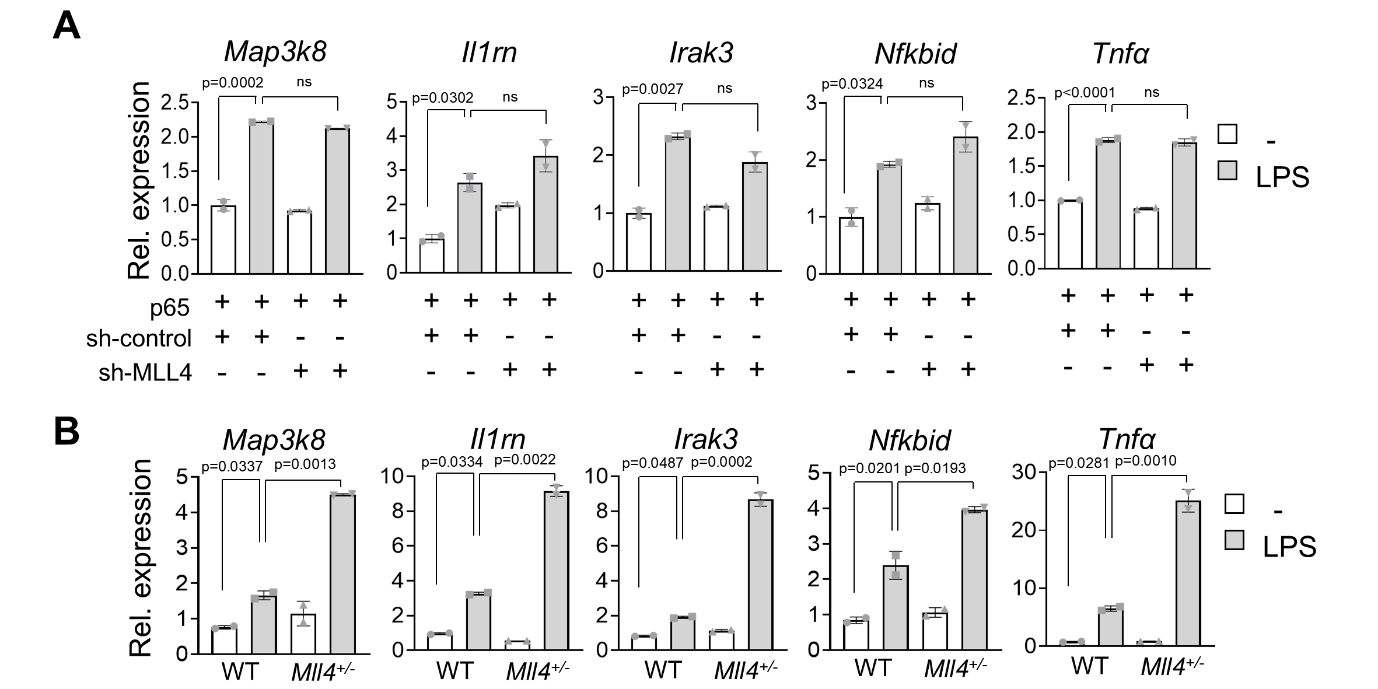


**Figure S2. Expression of NF-κB pathway genes in Hepa1c1c7 cells and primary mouse hepatocytes.** (A) mRNA levels of *Map3k8, Il1rn, Irak3, Nfkbid* and *Tnfα* were measured in Hepa1c1c7 cells transfected with sh-control, sh-MLL4, and p65, following vehicle (-) or LPS (100 ng/ml) treatment for 6 hours. (B) mRNA expression of *Map3k8, Il1rn, Irak3, Nfkbid* and *Tnfα* in primary hepatocytes from WT and *Mll4^+/-^* mice was assessed after LPS (100ng/ml) treatment for 6 hours (n=2 per group). Data represent the mean ± SD. Statistical differences were determined by two-way analysis of variance (ANOVA) with Tukey’s multiple comparisons test. ‘ns’ indicates no significant difference.


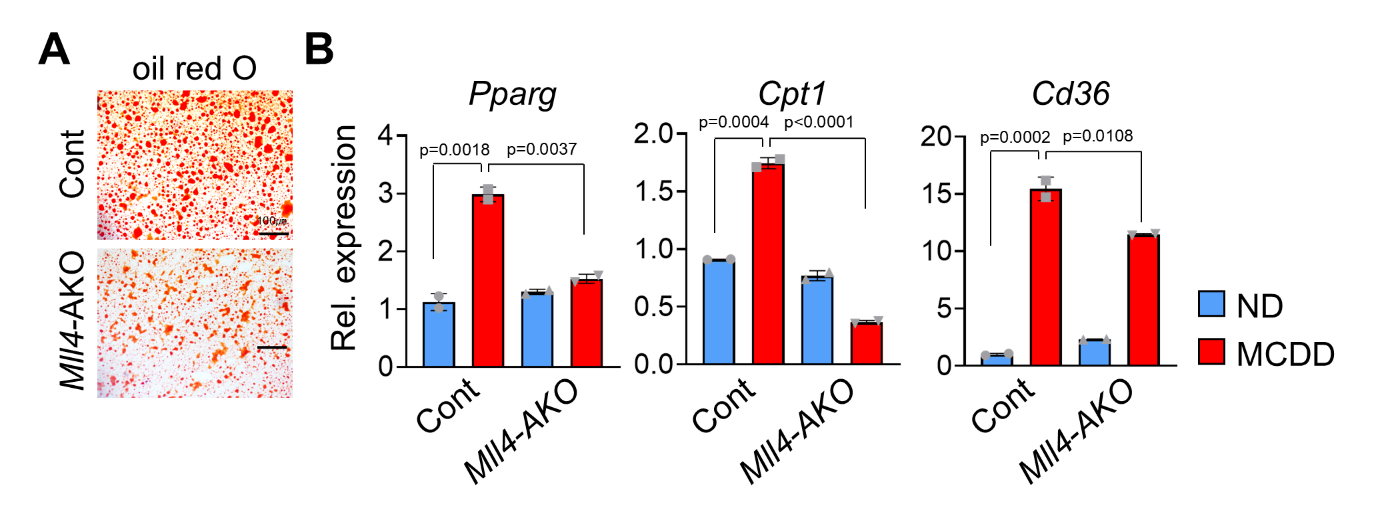


**Figure S3. Hepatocyte-specific deletion of *Mll4* prevents MCDD-induced steatosis.** (A) Oil red O staining of liver tissues from *Mll4^f/f^* and *Mll4*-AKO mice fed the MCDD. (B) Expression of liver steatosis marker genes in liver tissues was measured by qRT-PCR (n=2 per group). Results are presented as mean ± SD. Statistical differences were determined by two-way ANOVA with Tukey’s multiple comparisons test.


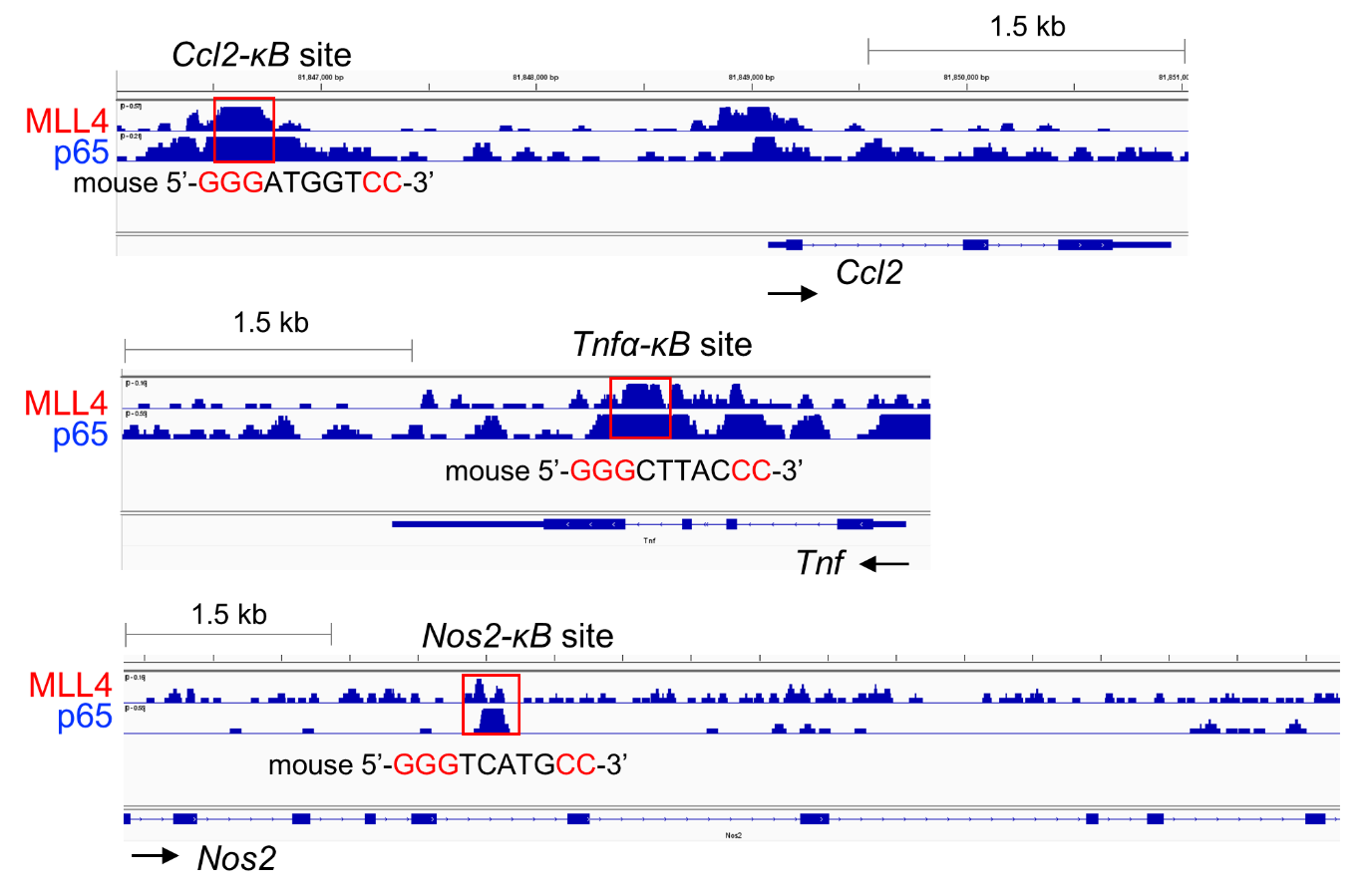


**Figure S4. A Schematic representation of ChIP-seq peaks for MLL4 and p65 at the *Ccl2, Tnfα* and *Nos2* loci.** MLL4 binding peaks in the *Tnfα* and *Nos2* genes were identified from the ChIP-seq dataset of bone marrow cells. Red-boxed regions highlight areas where MLL4 and p65 peaks overlap. The sequences of the p65 response element (κB site) in the mouse *Ccl2, Tnfα* and *Nos2* loci are shown.


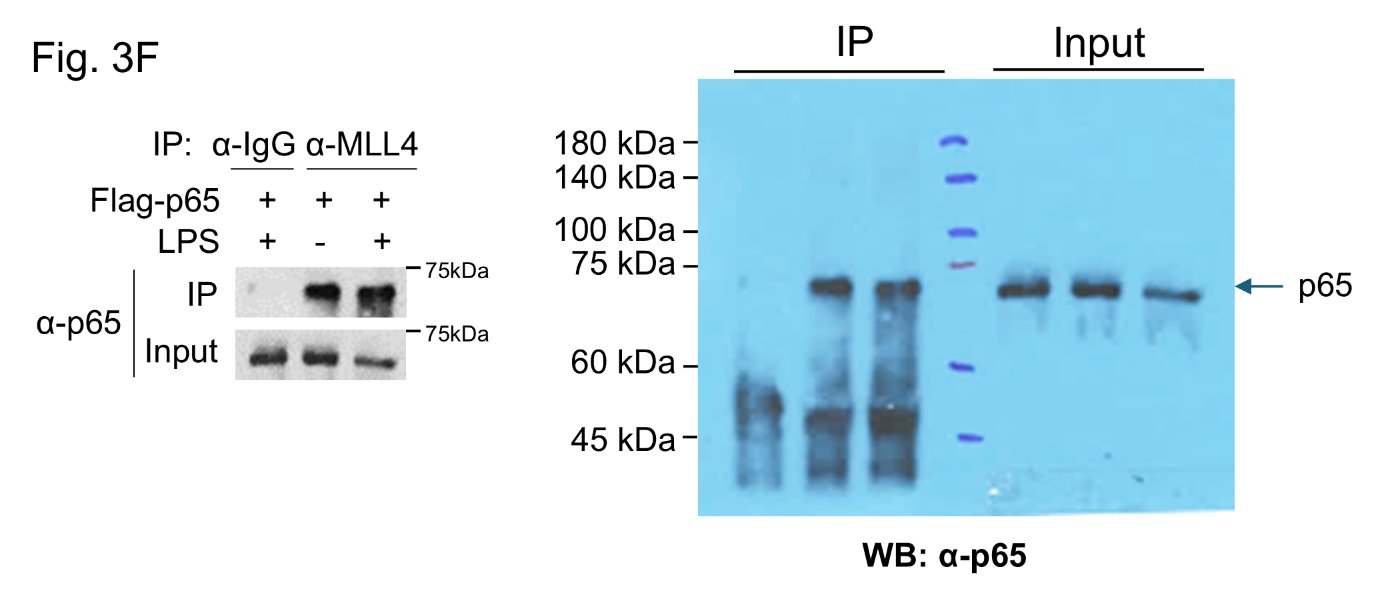


**Figure S5. Interaction between MLL4 and p65 in HepG2 cells.** HepG2 cells expressing Flag-p65 were subjected to immunoprecipitation with an anti-MLL4 antibody, followed by immunoblotting with an anti-p65 antibody. The results show that the interaction of p65 and MLL4 is enhanced by LPS treatment (cropped images are shown in Figure 3F).
